# Supplementary material for: Conditional chemoconnectomics (cCCTomics) as a strategy for efficient and conditional targeting of chemical transmission
Source: eLife. 2024 Apr 30;12:RP91927. doi: 10.7554/eLife.91927 (PMC11060718; doi:10.7554/eLife.91927)
Supplement: Figure 3—source data 3. [file elife-91927-fig3-data3.zip › Figure 3 source data3 part 1.zip!/Figure 3-Source data 3-part1/1063-13-CRISPResso_Report_8cz_fsdr.zip!/Nucleotide_percentage_quilt_around_sgRNA_CGTCGAGCACATGACATCAA.pdf]

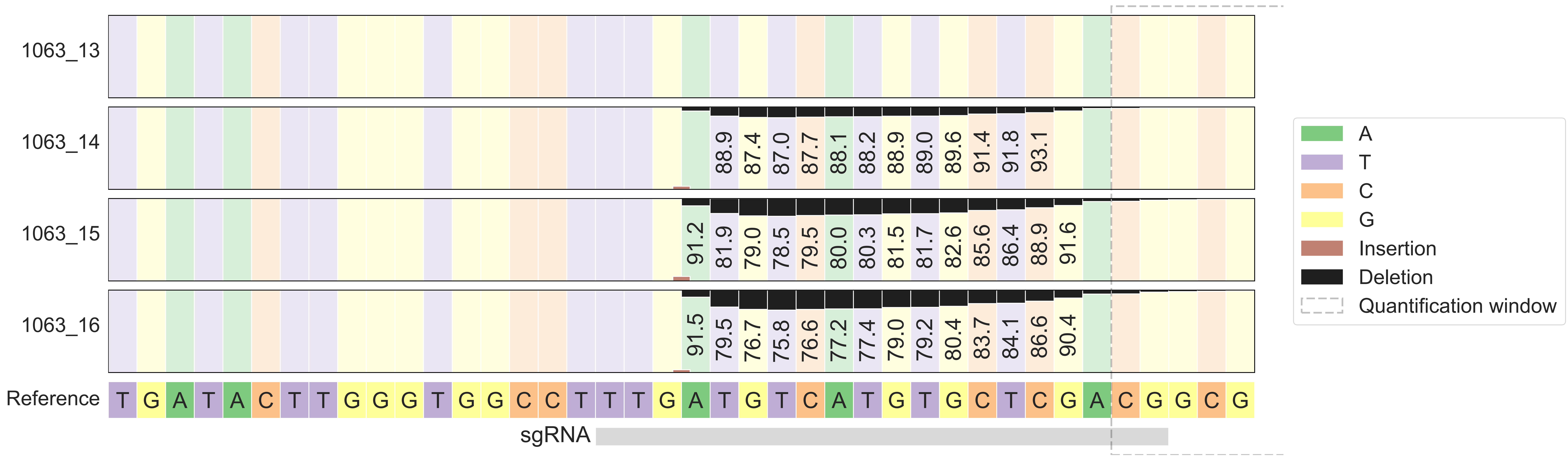
1063_13
1063_14
1063_15
1063_16
88.9 87.4 87.0 87.7 88.1 88.2 88.9 89.0 89.6 91.4 91.8 93.1
91.2 81.9 79.0 78.5 79.5 80.0 80.3 81.5 81.7 82.6 85.6 86.4 88.9 91.6
91.5 79.5 76.7 75.8 76.6 77.2 77.4 79.0 79.2 80.4 83.7 84.1 86.6 90.4
Reference
T G A T A C T T G G G T G G C C T T T G A T G T C A T G T G C T C G A C G G C G
sgRNA
A
T
C
G
Insertion
Deletion
Quantification window
